# Natriuretic peptide testing and heart failure diagnosis in primary care: diagnostic accuracy study

**DOI:** 10.3399/BJGP.2022.0278

**Published:** 2022-12-30

**Authors:** Clare J Taylor, José M Ordóñez-Mena, Sarah L Lay-Flurrie, Clare R Goyder, Kathryn S Taylor, Nicholas R Jones, Andrea K Roalfe, FD Richard Hobbs

**Affiliations:** Nuffield Department of Primary Care Health Sciences, University of Oxford, Oxford.; Nuffield Department of Primary Care Health Sciences, University of Oxford, Oxford.; Nuffield Department of Primary Care Health Sciences, University of Oxford, Oxford.; Nuffield Department of Primary Care Health Sciences, University of Oxford, Oxford.; Nuffield Department of Primary Care Health Sciences, University of Oxford, Oxford.; Nuffield Department of Primary Care Health Sciences, University of Oxford, Oxford.; Nuffield Department of Primary Care Health Sciences, University of Oxford, Oxford.; Nuffield Department of Primary Care Health Sciences, University of Oxford, Oxford.

**Keywords:** diagnosis, heart failure, natriuretic peptide, primary care, testing

## Abstract

**Background:**

Natriuretic peptide (NP) testing is recommended for patients presenting to primary care with symptoms of chronic heart failure (HF) to prioritise referral for diagnosis.

**Aim:**

To report NP test performance at European Society of Cardiology (ESC) and National Institute for Health and Care Excellence (NICE) guideline referral thresholds.

**Design and setting:**

Diagnostic accuracy study using linked primary and secondary care data (2004 to 2018).

**Method:**

The sensitivity, specificity, positive predictive value (PPV), and negative predictive value (NPV) of NP testing for HF diagnosis was assessed.

**Results:**

In total, 229 580 patients had an NP test and 21 102 (9.2%) were diagnosed with HF within 6 months. The ESC NT-proBNP threshold ≥125 pg/mL had a sensitivity of 94.6% (95% confidence interval [CI] = 94.2 to 95.0) and specificity of 50.0% (95% CI = 49.7 to 50.3), compared with sensitivity of 81.7% (95% CI = 81.0 to 82.3) and specificity of 80.3% (95% CI = 80.0 to 80.5) for the NICE NT-proBNP ≥400 pg/mL threshold. PPVs for an NT-proBNP test were 16.4% (95% CI = 16.1 to 16.6) and 30.0% (95% CI = 29.6 to 30.5) for ESC and NICE thresholds, respectively. For both guidelines, nearly all patients with an NT-proBNP level below the threshold did not have HF (NPV: ESC 98.9%, 95% CI = 98.8 to 99.0 and NICE 97.7%, 95% CI = 97.6 to 97.8).

**Conclusion:**

At the higher NICE chronic HF guideline NP thresholds, one in five cases are initially missed in primary care but the lower ESC thresholds require more diagnostic assessments. NP is a reliable ‘rule-out’ test at both cut-points. The optimal NP threshold will depend on the priorities and capacity of the healthcare system.

## INTRODUCTION

Heart failure (HF) affects around 1 million people in the UK and accounts for 3%–4% of NHS expenditure.^1^^,^^2^ Effective management improves quality and length of life for patients but establishing a diagnosis can be challenging because of the overlap of symptoms (such as breathlessness, exhaustion, or ankle swelling) with other conditions.^3^^–^^5^ Natriuretic peptides (NPs) are released by the myocardium in response to pressure or fluid overload and act on both the vasculature to relax smooth muscle and on the kidney to induce diuresis.^6^ NP levels are raised in people with HF and testing can aid diagnostic decision making. Guidelines recommend referral for cardiac imaging and specialist assessment depending on the NP level.^7^^–^^9^ Two types of NP test — B-type NP (BNP) and NT-proBNP — are currently available in clinical practice. BNP is biologically active and has a shorter half- life making it less stable over time.^6^

The European Society of Cardiology (ESC) and National Institute for Health and Care Excellence (NICE) recommend NP testing in both acute and chronic HF. The threshold values to rule out an acute HF diagnosis in an emergency department setting are consistent across guidelines (BNP <100 pg/mL and NT-proBNP <300 pg/mL) and supported by a large body of evidence.^7^^,^^10^ However, ESC and NICE guideline thresholds differ by more than threefold in chronic HF. These patients present to primary care with gradual onset of symptoms and NP testing is useful to inform the referral process. The ESC recommend referral for echocardiography and specialist assessment of BNP ≥35 pg/ mL or NT-proBNP ≥125 pg/ mL whereas NICE recommend a higher cut-off level of BNP ≥100 pg/mL or NT-proBNP ≥400 pg/mL.^7^^,^^8^ There is limited evidence on the optimal diagnostic NP threshold for primary care and current guidelines draw on small diagnostic accuracy studies, screening substudies, and consensus to make recommendations.^11^^–^^18^

The aim of this study was to report the real-world diagnostic performance of NP testing for chronic HF diagnosis at ESC and NICE referral thresholds.

## METHOD

A diagnostic accuracy study in a population- based cohort was conducted using linked electronic healthcare records. Primary care data from the Clinical Practice Research Datalink (CPRD) GOLD and Aurum databases were linked to inpatient Hospital Episodes Statistics (HES) admitted patient data and Index of Multiple Deprivation (IMD) socioeconomic data in England. Trends in NP testing have been published previously for this cohort.^9^ The combined CPRD databases contain data from over 1400 general practices in the UK, or 15.7% of the whole general practice population, and have been shown to be representative of the general population.^19^

**Table:** How this fits in

International guidelines recommend natriuretic peptide (NP) testing in primary care to prioritise referral for heart failure (HF) diagnostic assessment. European Society of Cardiology (ESC) and National Institute for Health and Care Excellence (NICE) guidelines differ significantly in their recommended NP referral threshold. The current study found at the lower ESC threshold fewer HF diagnoses were missed but more referrals from primary care would be required. Healthcare systems need to balance the risk of a missed or delayed diagnosis for individual patients with capacity in diagnostic services. An NP level below both the ESC and NICE thresholds was reliable in ruling out HF.

Patients aged ≥45 years in the two CPRD databases with an NP test result in their primary care record between 1 January 2004 and 31 December 2018 were included. Patients entered the cohort on the date of their NP test and exited the cohort on the date of their HF diagnosis or 6 months after their NP test date if they were not diagnosed with HF. Patients were only included if their primary care records were deemed acceptable for research purposes (a CPRD quality measure), eligible for linkage, and had been registered at a practice for ≥12 months. NP tests and HF diagnosis codes were identified in CPRD using clinical coding lists (see Supplementary Tables S1 and S2) derived from the NHS terminology and classifications browser and the Quality and Outcomes Framework guidance. Patients with a previous HF diagnosis were excluded.

### NP testing (index test)

NP level was analysed both as a continuous and a categorical variable using ESC (≥35 pg/mL and ≥125 pg/mL for BNP and NT-proBNP, respectively) and NICE (≥100 pg/mL and ≥400 pg/mL for BNP and NT-proBNP, respectively) referral thresholds for chronic HF diagnosis. NP test performance at the NICE thresholds for rapid referral (to be seen by a specialist within 2 weeks) of >400 pg/mL and >2000 pg/mL for BNP and NT-proBNP, respectively, were also explored.

### HF diagnosis (reference standard)

The primary outcome of HF diagnosis within 6 months of the most recent NP test was obtained from either a diagnostic code entered in the CPRD database or from HES Admitted Patient Care data based on hospital admission because of HF or echocardiography findings consistent with HF. HF diagnoses from primary care were also validated through data linkage with HES using International Classification of Diseases, 10th revision codes.

### Statistical analysis

Sociodemographic variables were summarised with median and interquartile range (IQR) for continuous variables, and frequencies and percentages for categorical variables. These were estimated overall, among participants with a NP test, and among those with and without HF.

Diagnostic accuracy for HF diagnosis was assessed by calculating sensitivity, specificity, positive predictive value (PPV) and negative predictive value (NPV), likelihood ratio, and diagnostic odds ratio using the ‘epitools’ package.^20^ Exact confidence intervals (CIs) for proportions were calculated using the binomial distribution. CIs for ratios were calculated using the Wald’s normal approximation. Receiver operating characteristic (ROC) curves were plotted for both tests to allow comparison of overall test performance between test types. The area under the ROC curve (AUC) was estimated using the ‘pROC’ package.^21^ All analyses were done in R (version 4.0.0) and used a 0.05 threshold to define statistical significance.

## RESULTS

A total of 229 580 patients had an NP test recorded in their primary care record with a median age of 61.2 years (IQR 52.0– 69.1), more females (57.5%, *n* = 132 002), and the majority were of White ethnicity (91.4%, *n* = 209 941) (Table 1). The median body mass index was 28.6 kg/m^2^ (IQR 25.1– 32.9), with a low-to-medium level of deprivation. Many participants were current or ex-smokers (66.7%, *n* = 117 521 and *n* = 35 676), had a diagnosis of hypertension (59.1%, *n* = 135 788), diabetes (24.7%, *n* = 56 782), or other cardiovascular disease (13.9%, *n* = 32 024).

**Table 1.** Sociodemographic characteristics and medical history of primary care patients aged ≥45 years overall, and with a BNP or NT-pro BNP test

**Characteristic**	**BNP test (*n* = 74 233)**	**NT-pro BNP test (*n* = 155 347)**	**Overall (*N* = 229 580)**
**Age, years, median (IQR)**	62.0 (53.0–70.0)	61.0 (52.0–69.0)	61.2 (52.0–69.1)

**Sex, female, *n* (%)**	42 538 (57.3)	89 464 (57.6)	132 002 (57.5)

**Ethnicity, *n* (%)**			
White	68 280 (92.0)	141 661 (91.2)	209 941 (91.4)
Indian	1195 (1.61)	2318 (1.49)	3513 (1.53)
Pakistani	608 (0.82)	1201 (0.77)	1809 (0.79)
Bangladeshi	102 (0.14)	352 (0.23)	454 (0.20)
Chinese	121 (0.16)	266 (0.17)	387 (0.17)
Other Asian	692 (0.93)	1094 (0.70)	1786 (0.78)
Black Caribbean	455 (0.61)	1756 (1.13)	2211 (0.96)
Black African	354 (0.48)	1251 (0.81)	1605 (0.70)
Other Black	148 (0.20)	706 (0.45)	854 (0.37)
Mixed	292 (0.39)	577 (0.37)	869 (0.38)
Other	596 (0.80)	1425 (0.92)	2201 (0.88)
Missing	1390 (1.87)	2740 (1.76)	4130 (1.80)

**BMI, kg/m^2^, median (IQR)**	28.4 (24.9–32.7)	28.7 (25.1–32.9)	28.6 (25.1–32.9)

**Smoking status, *n* (%)**			
Never	25 303 (34.1)	50 629 (32.6)	75 932 (33.1)
Former	37 807 (50.9)	79 714 (51.3)	117 521 (51.2)
Current	10 985 (14.8)	24 691 (15.9)	35 676 (15.5)
Missing	138 (0.19)	313 (0.20)	451 (0.20)

**IMD, quintile, *n* (%)**			
Q1 (least deprived)	21 096 (28.4)	30 085 (19.4)	51 181 (22.3)
Q2	16 822 (22.7)	32 861 (21.2)	49 683 (21.6)
Q3	15 665 (21.1)	31 593 (20.3)	47 258 (20.6)
Q4	13 374 (18.0)	31 187 (20.1)	44 561 (19.4)
Q5 (most deprived)	7260 (9.8)	29 527 (19.0)	36 787 (16.0)
Missing	16 (0.02)	94 (0.06)	110 (0.05)

**Medical history, *n* (%)**			
Diabetes	16 896 (22.8)	39 886 (25.7)	56 782 (24.7)
Hypertension	44 166 (59.5)	91 622 (59.0)	135 788 (59.1)
Atrial fibrillation	8822 (11.9)	17 403 (11.2)	26 225 (11.4)
Angina	6648 (8.96)	14 442 (9.30)	21 090 (9.19)
Ischaemic heart disease	8705 (11.7)	18 007 (11.6)	26 712 (11.6)
Myocardial infarction	4745 (6.39)	9729 (6.26)	14 474 (6.30)
Stroke	5678 (7.65)	12 629 (8.13)	18 307 (7.97)
Valvular disease	2949 (3.97)	5689 (3.66)	8638 (3.76)
Other CVD	10 586 (14.3)	21 438 (13.8)	32 024 (13.9)

**SBP, mmHg, median (IQR)**	136 (126–145)	136 (126–145)	136 (126–145)

**DBP, mmHg, median (IQR)**	78 (70–83)	78 (70–83)	78 (70–83)

**Total cholesterol, mmol/L, median (IQR)**	4.8 (4.0–5.6)	4.8 (4.0–5.6)	4.8 (4.0–5.6)

**BNP, pg/mL, median (IQR)**	61.1 (26.4–155.0)	—	61.1 (26.4–155.0)

**NT-proBNP, pg/mL, median (IQR)**	—	143.0 (60.0–413.0)	143.0 (60.0–413.0)

**Time between NP test and HF diagnosis**			
Days, median (IQR)	26 (8–66)	26 (7–64)	26 (7–65)
<2 weeks, *n* (%)	2313 (36.7)	5359 (35.5)	7672 (36.4)
2–6 weeks, *n* (%)	1634 (25.4)	3699 (25.1)	5333 (25.3)
≥6 weeks,*n* (%)	2570 (37.9)	5527 (39.4)	8097 (38.4)

*BMI = body mass index. BNP = B-type natriuretic peptide. CVD = cardiovascular disease. DBP = diastolic blood pressure. HF = heart failure. IMD = Index of Multiple Deprivation. IQR = interquartile range (25th and 75th percentiles). NP = natriuretic peptide. Q = quintile. SBP = systolic blood pressure.*

### NP testing

Two-thirds of all study participants had an NT-proBNP test (*n* = 155 347) and the other third had a BNP test (*n* = 74 233). BNP was the most common test until 2007, but since 2008 NT-proBNP has been more widely used (see Supplementary Table S3). There were no major differences between patients who underwent BNP versus NT-proBNP testing (Table 1), except those with a BNP test appeared to be more likely to come from a less deprived background.

### HF diagnosis

**Figure 1.**
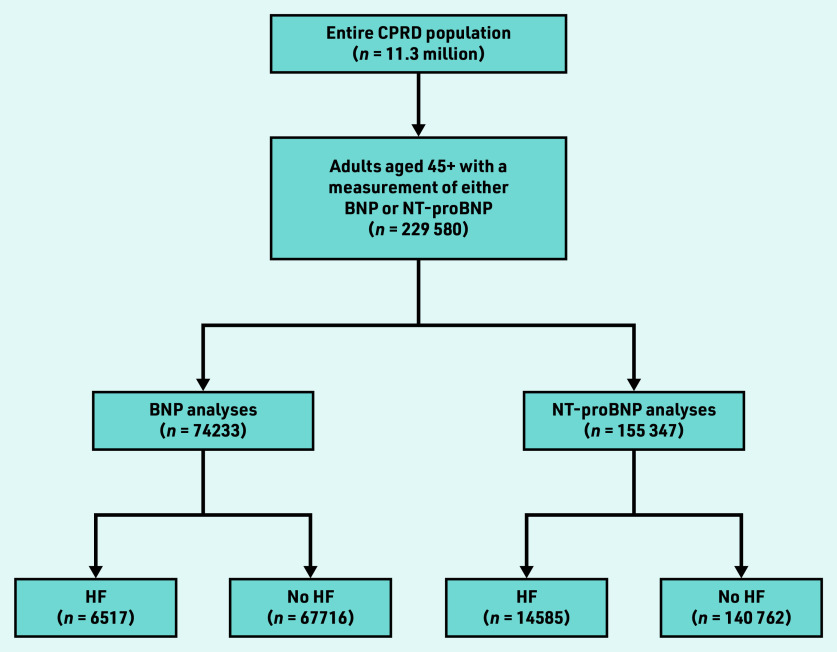
*Flowchart of patient inclusion. BNP = B-type NP. CPRD = Clinical Practice Research Datalink. HF = heart failure. NP = natriuretic peptide.*

Overall, 21 102 (9.2%) participants were diagnosed with HF within 6 months (Figure 1). Participants with HF were older than those without HF (median age 67 versus 61 years) and were more likely to be of White ethnicity (94.9%), current/ex-smokers (70.0%), with a history of hypertension (68.0%), diabetes (28.1%), or other cardiovascular diseases (21.0%) (Table 2).

**Table 2.** Sociodemographic characteristics and medical history of primary care patients aged ≥45 years with and without HF

**Characteristic**	**No HF (*n* = 208 478)**	**HF (*n* = 21 102)**
**Age, years, median (IQR)**	61.0 (52.0–69.0)	67.0 (60.0–74.0)

**Sex, female, *n* (%)**	121 668 (58.4)	10 334 (49.0)

**Ethnicity, *n* (%)**		
White	189 916 (91.1)	20 025 (94.9)
Indian	3318 (1.59)	195 (0.92)
Pakistani	1709 (0.82)	100 (0.47)
Bangladeshi	441 (0.21)	13 (0.06)
Chinese	362 (0.17)	25 (0.12)
Other Asian	1689 (0.81)	97 (0.46)
Black Caribbean	2075 (1.00)	136 (0.64)
Black African	1552 (0.74)	53 (0.25)
Other Black	814 (0.39)	40 (0.19)
Mixed	822 (0.39)	47 (0.22)
Other	1904 (0.91)	117 (0.55)
Missing	3876 (1.86)	254 (1.20)

**BMI, kg/m^2^, median (IQR)**	28.7 (25.1–32.9)	27.7 (24.2–32)

**Smoking status, *n* (%)**		
Never	69 650 (33.4)	6282 (29.8)
Former	105 967 (50.8)	11 554 (54.8)
Current	32 468 (15.6)	3208 (15.2)
Missing	393 (0.19)	58 (0.27)

**IMD, quintile, *n* (%)**		
Q1 (least deprived)	46 504 (22.3)	4677 (22.2)
Q2	44 914 (21.5)	4769 (22.6)
Q3	42 872 (20.6)	4386 (20.8)
Q4	40 551 (19.5)	4010 (19.0)
Q5 (most deprived)	33 546 (16.1)	3241 (15.4)
Missing	91 (0.04)	19 (0.09)

**Medical history, *n* (%)^a^**		
Diabetes	50 856 (24.4)	5926 (28.1)
Hypertension	121 447 (58.3)	14 341 (68.0)
Atrial fibrillation	20 143 (9.7)	6082 (28.8)
Angina	18 258 (8.76)	2832 (13.42)
Ischaemic heart disease	22 949 (11.0)	3763 (17.8)
Myocardial infarction	11 989 (5.75)	2485 (11.78)
Stroke	15 695 (7.53)	2612 (12.38)
Valvular disease	7233 (3.47)	1405 (6.66)
Other CVD	27 588 (13.2)	4436 (21.0)

**SBP, mmHg, median (IQR)**	136 (126–145)	135 (124–145)

**DBP, mmHg, median (IQR)**	78 (70–83)	76 (70–82)

**Total cholesterol, mmol/L, median (IQR)**	4.8 (4.1–5.6)	4.5 (3.7–5.3)

**BNP, pg/ml, median (IQR)**	54.0 (24.2–125.4)	335.9 (163.0–845.0)

**NT-proBNP, pg/ml, median (IQR)**	124.0 (56.0–306.0)	1358.0 (534.0–3230.0)

**Time between NP test and HF diagnosis**		
Days, median (IQR)	—	26 (7–65)
<6 weeks, *n* (%)		13 005 (61.6)
≥6 weeks,*n* (%)	—	8097 (38.4)

*BMI = body mass index; BNP = B-type NP. CVD = cardiovascular disease. DBP = Diastolic blood pressure. HF = heart failure. IMD = Index of multiple deprivation; IQR = interquartile range (25th and 75th percentiles). NP = natriuretic peptide. Q = quintile. SBP = systolic blood pressure.*

a *Percentages do not total 100 due to multimorbidity.*

NP testing was within 6 weeks of a confirmed HF diagnosis in 13 005 participants (61.6%), with 7672 participants (36.4%) diagnosed within 2 weeks overall. Participants with NT-proBNP >2000 pg/mL were more likely to be diagnosed within 2 weeks (44.9% compared with 35.4%, 24.8%, and 17.0% among those with 400–2000 pg/mL, 125–400 pg/mL, and <125 pg/ mL, respectively). This was similar for BNP >400 pg/mL, compared with lower levels of BNP (see Supplementary Table S4).

### Diagnostic test accuracy

The prevalence of HF in the populations tested with BNP and NT-proBNP was 8.8% and 9.4%, respectively. The median level of BNP was 336 pg/mL (IQR 163– 845) and NT-proBNP was 1358 pg/mL (IQR 534– 3230) among those diagnosed with HF (Table 2). The individual diagnostic test accuracy parameters for a HF diagnosis at both BNP and NT-proBNP referral thresholds are shown in Table 3.

**Table 3.** Diagnostic test accuracy parameters for the diagnosis of HF using BNP and NT-proBNP level NICE and ESC referral thresholds^a^

**Test**	**BNP (total *N*= 74 233, HF *n* = 6517, 8.8%)**			**NT-proBNP (total *N*= 155 347, HF *n*= 14 585, 9.4%)**		
	**Cut off, ≥35 pg/mL**	**Cut off, ≥100 pg/mL**	**Cut off, >400 pg/mL**	**Cut off, ≥125 pg/mL**	**Cut off, ≥400 pg/mL**	**Cut off, >2000 pg/mL**
**TP, *n***	6313	5769	2909	13 801	11 913	5674
**FN, *n***	204	748	3608	784	2672	8911
**FP, *n***	43 793	20 942	4510	70 365	27 788	5424
**TN, *n***	23 923	46 774	63 206	70 397	112 974	135 338
**Sensitivity, % (95% CI)**	96.9 (96.4 to 97.3)	88.5 (87.7 to 89.3)	44.6 (43.4 to 45.9)	94.6 (94.2 to 95.0)	81.7 (81.0 to 82.3)	38.9 (38.1 to 39.7)
**Specificity, % (95% CI)**	35.3 (35.0 to 35.7)	69.1 (68.7 to 69.4)	93.3 (93.1 to 93.5)	50.0 (49.7 to 50.3)	80.3 (80.0 to 80.5)	96.1 (96.0 to 96.2)
**PPV, % (95% CI)**	12.6 (12.3 to 12.9)	21.6 (21.1 to 22.1)	39.2 (38.1 to 40.3)	16.4 (16.1 to 16.6)	30.0 (29.6 to 30.5)	51.1 (50.2 to 52.1)
**NPV, % (95% CI)**	99.2 (99.0 to 99.3)	98.4 (98.3 to 98.5)	94.6 (94.4 to 94.8)	98.9 (98.8 to 99.0)	97.7 (97.6 to 97.8)	93.8 (93.7 to 93.9)
**LR+ (95% CI)**	1.50 (1.49 to 1.51)	2.86 (2.82 to 2.90)	6.70 (6.45 to 6.97)	1.89 (1.88 to 1.91)	4.14 (4.08 to 4.19)	10.1 (9.77 to 10.44)
**LR– (95% CI)**	0.09 (0.08 to 0.10)	0.17 (0.16 to 0.18)	0.59 (0.58 to 0.61)	0.11 (0.10 to 0.12)	0.23 (0.22 to 0.24)	0.64 (0.63 to 0.64)
**DOR (95% CI)**	16.89 (14.72 to 19.5)	17.22 (15.94 to 18.63)	11.30 (10.67 to 11.97)	17.61 (16.38 to 18.94)	18.12 (17.35 to 18.93)	15.89 (15.22 to 16.59)

a *The overall number of participants (total N) with either test was different, therefore also the number with HF, as well as the prevalence (%). BNP = B-type NP. DOR = diagnostic odds ratio. ESC = European Society of Cardiology. FN = false negative. FP = false positive. HF = heart failure. LR = likelihood ratio. NICE = National Institute for Health and Care Excellence. NP = natriuretic peptide. NPV = negative predictive value. PPV = positive predictive value. TN = true negative. TP = true positive.*

The lower ESC referral thresholds of ≥35 pg/mL for BNP and ≥125 pg/ mL for NT-proBNP had sensitivities of 96.9% (95% CI = 96.4 to 97.3) and 94.6% (95% CI = 94.2 to 95.0), and specificities of 35.3% (95% CI = 35.0 to 35.7) and 50.0% (95% CI = 49.7 to 50.3), respectively (Table 3). PPV for NP testing was 12.6% (95% CI = 12.3 to 12.9) for BNP and 16.4% (95% CI = 16.1 to 16.6) for NT-proBNP, and NPVs of 99.2% (95% CI = 99.0 to 99.3) and 98.9% (95% CI = 98.8 to 99.0), were found for BNP and NT-proBNP, respectively.

At the higher NICE referral threshold of ≥100 pg/ml for BNP and ≥400 pg/ml for NT-proBNP, sensitivity was 88.5% (95% CI = 87.7 to 89.3) and 81.7% (95% CI = 81.0 to 82.3), specificity was 69.1% (95% CI = 68.7 to 69.4) and 80.3% (95% CI = 80.0 to 80.5), PPV was 21.6% (95% CI = 21.1 to 22.1) and 30.0% (95% CI = 29.6 to 30.5), and NPVs were 98.4% (95% CI = 98.3 to 98.5) and 97.7% (95% CI = 97.6 to 97.8), respectively (Table 3).

**Figure 2.**
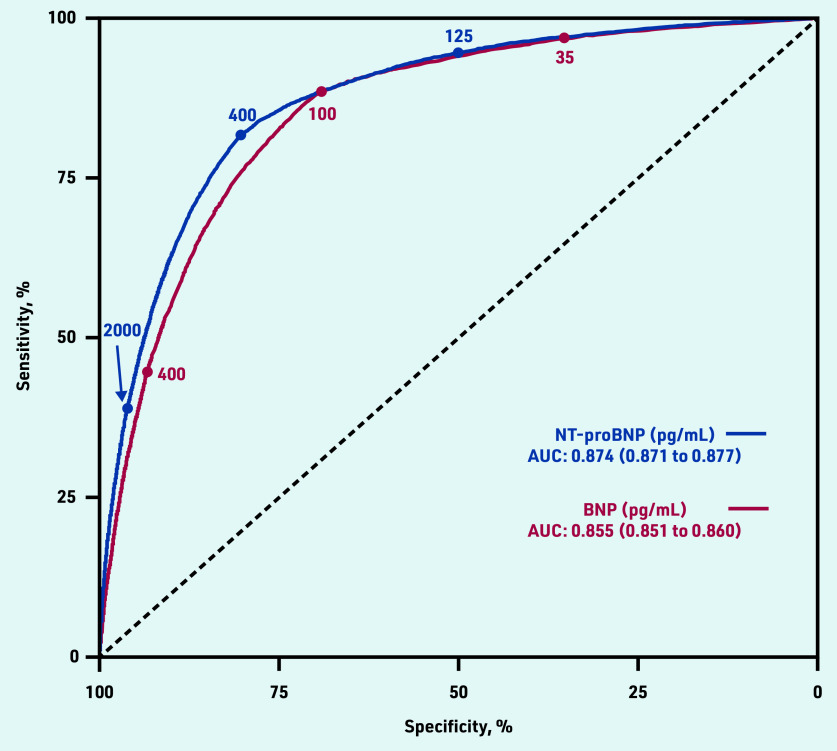
*ROC curve for HF diagnosis for BNP and NT-proBNP tests at ESC and NICE referral thresholds. AUC = area under the ROC curve. BNP = B-type NP. ESC = European Society of Cardiology. HF = heart failure. NICE = National Institute for Health and Care Excellence. NP = natriuretic peptide. ROC = receiver operating characteristic.*

In summary, there was a clinically meaningful difference in all the key test performance measures between the ESC and NICE guideline recommended cut- off levels. As expected at the higher NICE threshold, specificity was much better (80.3% versus 50.0%) but at the expense of a lower sensitivity (81.7% versus 94.6%) for NT-proBNP (Table 3). Figure 2 shows the sensitivity and specificity for possible values of BNP and NT-proBNP. There was a small but statistically significant improvement in overall performance using NT-proBNP compared with BNP (AUC 0.874, 95% CI = 0.871 to 0.877) for NT-proBNP versus 0.855 (95% CI = 0.851 to 0.860) for BNP.

## DISCUSSION

### Summary

In this large, real-world diagnostic accuracy study in 229 580 primary care patients who underwent NP testing, the ESC chronic HF referral threshold of NT-proBNP ≥125 pg/mL had a high sensitivity (94.6%, 95% CI = 94.2 to 95.0), but low specificity (50.0%, 95% CI = 49.7 to 50.3) compared with a moderate sensitivity (81.7%, 95% CI = 81.0 to 82.3) and moderate specificity (80.3%, 95% CI = 80.0 to 80.5) for the NICE NT-proBNP ≥400 pg/mL referral threshold. At the current NICE threshold, one in five cases of HF are initially missed by NP testing; however, for each additional new HF diagnosis at the lower ESC cut-off, around 20 extra patients require diagnostic assessment. Both guidelines accurately ruled out HF (NPVs for ESC of 98.9%, 95% CI = 98.8 to 99.0 and NICE 97.7%, 95% CI = 97.6 to 97.8). Better diagnostic performance was shown for NT-proBNP than BNP.

### Strengths and limitations

A strength of using real-world patient data to evaluate diagnostic test accuracy is a larger sample size than would routinely be used in cross-sectional studies, and this is especially important for diseases of lower prevalence.^22^ The largest prospective diagnostic accuracy studies of NP testing to identify people with HF in primary care had <750 participants.^7^^,^^8^^,^^13^ The current study included 229 580 patients with an NP test over a 14-year period.

Routinely collected data do have limitations as the reference standard is not assessed for research purposes or measured in everyone. Instead, a comprehensive clinical coding list was used to determine the presence or absence of a HF diagnosis.^23^ Assuming GPs in the UK follow NICE guidelines then those with a primary care NP test above the lower ESC cut-off but below NICE would not be referred for diagnostic assessment, but greater sensitivity was observed at the lower ESC level, suggesting HF diagnosis was made through a variety of routes. However, some people who did have HF may have been missed and, if so, false negatives may be underestimated and the sensitivities overestimated.

Baseline NP levels may have been affected by population characteristics such as age, HF symptoms, medications (for example, diuretics and renin–angiotensin system antagonists), and other medical conditions including chronic kidney disease and atrial fibrillation.^24^ These covariates were not stratified for as the aim was to explore test accuracy for guideline referral thresholds, and both ESC and NICE do not currently have differing thresholds based on other factors. The current study is also most relevant to healthcare systems with a strong primary care base where NP testing is required before referral for specialist diagnostic assessment. The way that HF is defined has changed over time, with a greater emphasis in the recent ESC 2021 guidelines on the importance of elevated NP levels when making a diagnosis.^7^ This may have resulted in a change to the classification of some people in the early years of the study if these contemporary HF definitions were used. There were also insufficient data available on left ventricular ejection fraction to report these results by HF classification (HF with reduced versus preserved ejection fraction).

### Comparison with existing literature

The current results using primary care data are consistent with the findings of a large systematic review of NP testing for chronic HF across ambulatory settings, published in 2018.^18^ The review included 39 diagnostic accuracy studies of NP testing, five of which were conducted in primary care and reported a pooled sensitivity of 95% (95% CI = 90 to 98) for the BNP ≥100 pg/mL referral threshold, very similar to the current study, but with no difference between BNP and NT-proBNP test performance overall. For primary care studies that used NT-proBNP testing, sensitivity was 99% (95% CI = 57 to 100) and specificity was 60% (95% CI = 44 to 74) but these were limited to only two small studies. The current study included >20 000 patients with a new HF diagnosis and is therefore likely to be more representative of NP test performance in NHS primary care.

The current study showed significantly lower sensitivities for BNP and NT-proBNP at the NICE referral thresholds than those reported for patients with acute HF in a large systematic review and meta- analysis of diagnostic accuracy studies in the acute care setting.^25^ However, the NT-proBNP threshold was 300 pg/mL in this meta- analysis, so a higher sensitivity is expected, and the diagnostic accuracy of a test is also greater in settings where patients have more severe symptoms. Furthermore, the review only included original studies from patients admitted to hospital with acute HF and the prevalence of HF was much greater (ranging from 23% to 82%) than in the current primary care- based study (∼9%).

Using real-world patient data to evaluate diagnostic test accuracy is an established methodology that has been used in cancer diagnostics in primary care. CPRD data have been used to examine the diagnostic performance of the biomarker cancer antigen 125 that is frequently measured in females who present to their primary care physician with symptoms that could be caused by ovarian cancer and these data were used to estimate probability in females of different ages.^26^ Routinely collected data have also been used to evaluate the test accuracy of faecal calprotectin at different thresholds in the diagnosis of inflammatory bowel disease.^27^

### Implications for research and practice

The current study suggests that at the current NICE threshold, one in five cases are initially missed following NP testing in primary care and diagnosed through an alternative route within 6 months. However, for every additional case of HF detected at the lower ESC threshold, around 20 extra patients would need to be referred for echocardiography and specialist assessment. The optimal NP threshold for referral for HF diagnosis will therefore depend partly on capacity within the healthcare setting. Patients with very high NP levels have worse outcomes and require timely referral for diagnosis and treatment initiation, and this could be hampered if diagnostic services are overwhelmed by a lower NP referral threshold.^28^ In the current study, an NP value below either the ESC or NICE thresholds effectively ruled out HF and this can be extremely valuable to aid diagnostic decision making in primary care.^29^ This trade-off between ensuring capacity in out-patient clinics is not breached versus missing a diagnosis of HF is challenging for both the referring primary care clinician and the HF specialist. Where the patient only has mild symptoms and an NP level below the threshold, monitoring in primary care may be necessary.

The current study also found that people with risk factors (ex-smoker, hypertension, high cholesterol) and established cardiovascular disease were more likely to receive an NP test, suggesting clinicians are targeting testing at those felt to be at increased risk of HF.^30^ In practice, NP levels are dynamic and will vary in relation to a range of factors, including weight status, renal function, age, and atrial fibrillation. Although current ESC and NICE guidelines have maintained the current NP thresholds used in this analysis, a recent position paper from the ESC on the use of NP testing suggested some of these variables, particularly obesity, should be considered when interpreting results.^7^^,^^8^^,^^31^ Further research could help refine referral thresholds for these key subgroups to help inform future guidance. In the meantime, clinicians in primary care should be aware that if higher NP thresholds for specialist assessment are recommended in national guidelines, patients with results that fall just below this level may still require referral for diagnostic assessment. Repeat testing of NP results over time in patients who are symptomatic may also help improve the sensitivity of testing and help minimise delays in HF diagnosis.

## Data

Data for this study were obtained on licence from CPRD and cannot be shared. Equivalent data can be obtained directly from CPRD with relevant ISAC approval. Hospital Episode Statistics and Office for National Statistics data are subject to copyright © (2018) and can be re-used with the permission of The Health and Social Care Information Centre. All rights reserved.
